# From ‘Emotional Storm’ to Post-Traumatic Growth Among Caregivers of Individuals with Mental Illness and Substance Use Disorders: An Integrative Review

**DOI:** 10.3390/nursrep16080280

**Published:** 2026-08-11

**Authors:** Pimwalunn Aryuwat, Jessica Holmgren

**Affiliations:** 1 Boromarajonani College of Nursing Udonthani, Faculty of Nursing, Praboromarajchanok Institute, Udon Thani 41330, Thailand; pimwalunn.aryuwat@bcnu.ac.th; 2 Department of Health Sciences, Innovation and Design, Mälardalen University, Hamngatan 15, 631 05 Eskilstuna, Sweden

**Keywords:** caregiver resilience, post-traumatic growth, family caregiving, mental illness, substance use disorders, psychological adaptation

## Abstract

**Background/Objective:** Family caregivers of individuals with serious mental illness and substance use disorders experience substantial psychological burden. However, emerging evidence suggests many develop resilience and post-traumatic growth despite adversity. This integrative review synthesizes evidence on resilience, coping strategies, post-traumatic growth, and protective factors among these caregivers. **Methods:** The Whittemore and Knafl integrative review framework guided this synthesis. A systematic literature search was conducted across PubMed, CINAHL, and PsycINFO databases for peer-reviewed articles examining resilience, coping mechanisms, and post-traumatic growth in family caregivers of individuals with serious mental illness and substance use disorders. Qualitative, quantitative, and mixed-methods studies were included. Study quality was assessed using the Mixed Methods Appraisal Tool (MMAT). Data were extracted and analyzed using thematic synthesis to identify common patterns. **Results:** Multiple protective factors facilitate resilience and post-traumatic growth, including cultural values, family connectedness, religious faith, and emotional intelligence. Individual characteristics such as personality traits and decision-making styles influence adaptation outcomes. Structured interventions including psychoeducation, mindfulness practices, and peer support show initial evidence of enhancing caregiver resilience and psychological well-being, based on a single randomized controlled trial and cross-sectional associations. The caregiving experience provides opportunities for meaningful personal transformation despite significant burden. **Conclusions:** Family caregivers of individuals with serious mental illness and substance use disorders possess substantial capacity for positive psychological change. Healthcare professionals should implement strength-based, family-centered interventions acknowledging caregiver resilience. Future research employing longitudinal designs and investigating intervention mechanisms is essential to advance caregiver-focused mental health services.

## 1. Introduction

Serious mental illness (SMI) and substance use disorders (SUD) represent a substantial global health burden, affecting more than one billion individuals and accounting for approximately 19% of global disability burden [1]. These conditions are characterized by prolonged illness courses, episodic crises, and variable treatment responses, requiring sustained engagement from family caregivers who assume responsibility for daily functioning, symptom management, and psychosocial support [2]. The progressive and often unpredictable nature of these disorders creates a complex caregiving environment in which family members navigate multiple roles and responsibilities at the same time, often extending over decades [3].

Family caregivers of individuals with SMI and SUD experience substantial burden—the cumulative physical, emotional, social, and financial strain resulting from the caregiving role [4]. Research indicates that among caregivers of patients with psychoactive substance use disorders, a large proportion experience concurrent depressive and anxiety symptoms, with 55.83% reporting moderate to severe depression and 34.2% reporting moderate to severe anxiety [5]. Beyond measurable burden, caregivers navigate unpredictable emotional traces characterized by fear, uncertainty, and grief, often increased by the instability inherent in mental health crises and substance use relapse cycles [6]. The caregiving experience is further intensified by stigma surrounding both mental illness and addiction, in which family members frequently internalize societal stigma, a phenomenon conceptualized as “courtesy stigma”, which combines psychological distress and limits social support mobilization [7,8]. Additionally, caregivers challenge with role conflict, disrupted life courses, and persistent concerns regarding the safety of their loved ones, collectively placing them in a state of continuous care with limited opportunities for personal development or self-care [9,10].

Nevertheless, emerging evidence reveals an alternative and important perspective that challenges the primarily deficit-focused narrative surrounding family caregiving. Despite sustained adversity and significant burden, many family caregivers develop resilience and experience post-traumatic growth (PTG), positive psychological change resulting from struggling with challenging life circumstances [11]. Resilience, conceptualized as an adaptive process utilizing personal and environmental resources, serves as a critical mediator between caregiving stress and psychological well-being outcomes [12]. Research demonstrates that cultural values such as family connectedness and religious faith serve as powerful protective factors facilitating psychological adaptation and growth [13]. Furthermore, emotional intelligence also appears to help caregivers cope with distress more adaptively, as those with stronger capabilities manage to develop more flexible responses to the demands of caregiving [14]. This protective role can be further situated within the clinical neuroscience literature on affect regulation, which indicates that impaired emotional processing capacity, such as alexithymia, is associated with high psychiatric symptom burden, including depressive symptoms and increased suicidal ideation, among individuals exposed to chronic psychological strain [15]. Regarding the caregiving context, this shared connection suggests that caregivers with lessened ability to identify and articulate their emotional states may be more vulnerable to the cumulative psychiatric condition of sustained caregiving responsibilities, whereas well-regulated affective processing may buffer against this risk and support adaptive engagement with the caregiving role [16]. Structured interventions, including psychoeducation, mindfulness, and peer support, have shown promise in strengthening this resilience and improving caregivers’ overall psychological well-being [17]. These findings point to caregiving as an experience that, despite its difficulties, can open pathways toward personal growth and psychological transformation.

### Problem Identification

While individual studies have examined resilience, coping strategies, and post-traumatic growth in caregiving populations, the field lacks a comprehensive synthesis of how these phenomena are evident specifically within serious mental illness and substance use disorder caregiving contexts. Prior review has focused on caregiver burden, distress, and targeted interventions, overlooking the processes and contextual factors (culture, individual traits, family dynamics, structural resources) that enable resilience and post-traumatic growth in this population [18].

Mental health nurses work closely with both patients and their families, often being the first to notice when family caregivers are struggling [19]. Most studies on how caregivers bounce back and grow from difficult experiences have been written for a broad healthcare audience, without really exploring what this means for nurses specifically [20]. This matters because nurses are well positioned to assess how caregivers are coping, support their strengths, and connect them with appropriate help [21]. This integrative review therefore aims not only to synthesize the available evidence, but also to draw out what it means for mental health nurses working with families affected by serious mental illness and substance use disorders. The primary aim of this integrative review is to synthesize existing empirical evidence regarding resilience, coping strategies, post-traumatic growth, and protective factors among family caregivers of individuals with serious mental illness and substance use disorders.

Specifically, the review addresses the following research questions: (1) What are the primary sources of stress and burden experienced by family caregivers of individuals with SMI and SUD, and how do caregivers appraise and respond to these stressors? (2) What individual, familial, social, and contextual factors facilitate resilience and post-traumatic growth among family caregivers in this population? (3) What evidence-based interventions effectively enhance resilience, capacity, and psychological well-being in family caregivers, and through what mechanisms do these interventions apply their effects? This synthesis is intended to support mental health professionals move beyond deficit-focused models toward family-centered, resilience-promoting care, and to inform policy and future research priorities in this area.

## 2. Materials and Methods

The Whittemore and Knafl framework was selected to guide this integrative review [22] since the primary goal was to synthesize existing evidence regarding resilience, coping strategies, post-traumatic growth, and protective factors among family caregivers of individuals with serious mental illness and substance use disorders, consistent with the aim and research questions set out in Section 1. This framework has five stages: problem identification, literature search, data evaluation, data analysis, and presentation. Employing the Whittemore and Knafl framework allowed for the inclusion of diverse methodologies, thereby providing a comprehensive view of available evidence. Additionally, the Preferred Reporting Items for Systematic Reviews and Meta-Analyses (PRISMA) 2020 statement [23] informed the reporting of results to ensure transparency and methodological rigor. (see completed checklist in Supplementary Materials).

### 2.1. Literature Search Strategy

A systematic literature search was conducted following consensus among all team members regarding the search strategy. The search was performed across multiple electronic databases including PubMed, CINAHL, and PsycINFO to ensure comprehensive coverage of the nursing, psychology, and health sciences literature. Advanced keyword searching was employed across databases using search terms tailored to each database’s taxonomy, with the following core search terms: (caregiver* OR “family member*” OR “informal caregiver*”) AND (resilience OR “post-traumatic growth” OR coping OR adaptation OR “psychological well-being”) AND (mental illness OR psychosis OR schizophrenia OR “bipolar disorder” OR depression) AND (burden OR stress OR “protective factor*”). Following the electronic databases search, supplementary hand-searching of reference lists of relevant articles and personal libraries was conducted to identify additional sources.

### 2.2. Inclusion and Exclusion Criteria

Inclusion and exclusion criteria were developed collaboratively among team members based on the review’s stated aim. No specific time restrictions were imposed to ensure comprehensive synthesis of available evidence. Studies were included if: (1) the study examined resilience, coping strategies, or post-traumatic growth in family caregivers; (2) participants included family members or informal caregivers of individuals with serious mental illness and/or substance use disorders; (3) the study employed quantitative, qualitative, or mixed methods designs; and (4) findings were published in peer-reviewed journals in the English language. Studies were excluded if: (1) the study focused exclusively on healthcare providers or professional caregivers; (2) participants were institutional caregivers rather than family members; (3) the study examined interventions without assessment of resilience or related outcomes; and (4) the publication was a review article, opinion piece, or non-empirical work. Family caregivers were defined as spouses, children, parents, siblings, or other relatives, or close friends providing informal care to individuals with SMI or SUD. The review was deliberately restricted to informal, family-based caregivers rather than paid or professional caregivers because the constructs under investigation, resilience and post-traumatic growth, are conceptually grounded in the experience of personal, relational transformation following exposure to a loved one’s adversity, a process that is qualitatively distinct from the occupational stress and burnout experienced by paid care staff, who enter the caregiving role voluntarily, within defined professional boundaries, and with compensation. In contrast, family caregivers undertake this role without formal preparation, choice, or payment, and remain embedded within the ongoing relational and family system of the person for whom they care; it is involuntary, relationally embedded form of caregiving that the review sought to characterize.

### 2.3. Data Evaluation and Quality Assessment

Title and abstract screening were conducted by two reviewers. Any disagreements regarding eligibility were resolved through discussion between the two reviewers to reach a consensus. Two reviewers independently evaluated full-text articles, with disagreements resolved through consensus discussion. Data extraction was performed using a standardized template collaboratively developed by the research team. The template was piloted on two of the included studies to confirm its comprehensiveness and clarity before being applied to the full set of included studies, with minor refinements made accordingly. Two reviewers independently extracted data from each included study; extracted data were then cross-checked, and any discrepancies were resolved through discussion between the two reviewers until consensus was reached. The template captured study design, participant characteristics, the specific method or instrument used to assess resilience, coping, or post-traumatic growth, interventions when applicable, and principal findings.

Across the included quantitative studies, these constructs were operationalized using validated, standardized self-report instruments: Mirhosseini et al. [24] and Wu et al. [25] both administered the Connor-Davidson Resilience Scale (CD-RISC); Wu et al. [25] additionally administered the Posttraumatic Growth Inventory (PTGI), the Simplified Coping Style Questionnaire, and the Perceived Social Support Scale; Mirhosseini et al. [24] additionally administered the Revised Life Orientation Test (LOT-R) to assess optimism; and Stjernswärd and Hansson [26] administered the Five Facet Mindfulness Questionnaire, the Self-Compassion Scale-Short Form, and the Perceived Stress Scale as pre-/post-intervention outcome measures.

Two further quantitative studies Akinwale et al. [27] and Mihan et al. [10] assessed related constructs, namely emotional intelligence, psychological distress, well-being, and caregiver burden, using validated scales (including the Zarit Burden Interview), but did not directly administer a resilience, coping, or post-traumatic growth instrument. Across the included qualitative studies, these constructs were instead elicited through semi-structured or in-depth interviews (Maina et al. [28]; Peng et al. [29]; Villena Jimena et al. [30]), hermeneutic secondary analysis of pre-existing interview transcripts (Kalhovde and Kitzmüller [6]), interpretative phenomenological analysis of telephonic interviews (Selotole et al. [31]), or thematic analysis of published first-person written accounts (Estradé et al. [32]), without a priori standardized measurement. The specific instrument or interview-based method used in each individual study is detailed in Table 1. Data evaluation and quality assessment were conducted independently by the two reviewers to ensure methodological rigor throughout the review process.

Study quality was assessed using the Mixed Methods Appraisal Tool (MMAT) [33], a validated instrument designed to evaluate the methodological quality of quantitative, qualitative, and mixed methods studies within a single appraisal process. The MMAT comprises a set of criteria organized by study design type, with each design having five quality criteria. Two reviewers independently assessed each study using the MMAT, with disagreements resolved through discussion between the two reviewers until consensus was reached. For this review, studies meeting all five quality criteria were categorized as high quality, those meeting four criteria were categorized as moderate quality, and those meeting three or fewer criteria were categorized as low quality. Quality scores were documented in summary tables alongside study characteristics and were considered during data synthesis and interpretation.

### 2.4. Data Analysis and Synthesis

Data analysis employed a thematic synthesis approach informed by the framework of Braun and Clarke [34] to organize and interpret findings across diverse studies, rather than a quantitative meta-analytic approach such as pooled effect-size estimation with forest plots. This choice reflected the substantial heterogeneity of study designs (qualitative, cross-sectional quantitative, and a single randomized controlled trial), the use of non-comparable outcome measures across studies, and the inclusion of only one intervention trial, none of which met the minimum conditions of design and outcome homogeneity required for statistically meaningful pooling. Thematic synthesis was therefore selected because it provides a systematic and rigorous method for identifying, analyzing, and reporting patterns within qualitative and mixed-methods data, and permits integration of diverse research designs consistent with the overarching Whittemore and Knafl integrative review framework. Extracted data were coded and categorized into four primary domains: (1) individual-level protective factors such as emotional intelligence, optimism, and meaning-making; (2) familial and social resources including family solidarity, peer support, and cultural values; (3) structural and systemic factors such as access to services and policy support; and (4) intervention mechanisms encompassing psychoeducation, mindfulness practices, and cognitive reappraisal techniques. Within each domain, findings were further organized by study design and population characteristics, including geographical region, cultural context, and type of mental illness or substance use disorder, to identify patterns and variations in how resilience manifests across different contexts. Summary tables were developed to present study characteristics, participant demographics, key resilience factors, and main findings, enabling systematic comparison across studies. Narrative synthesis was subsequently employed to interpret patterns, synthesize findings, and identify evidence gaps in the existing literature. During the synthesis process, quality assessment scores were considered, with particular emphasis placed on findings derived from moderate and high-quality studies to ensure the reliability and validity of conclusions.

## 3. Results

### 3.1. Search Results and Study Selection

**Figure 1:**
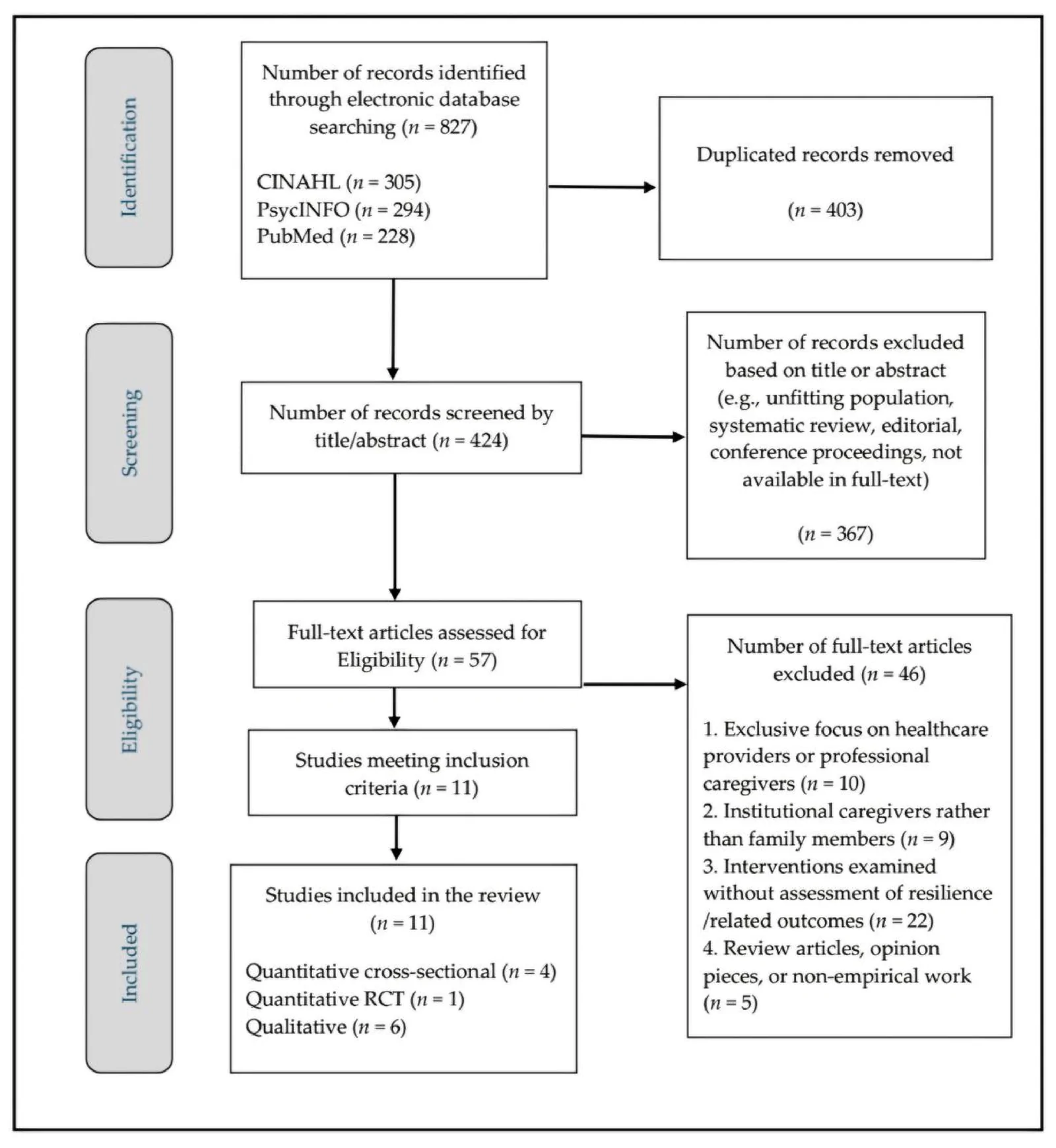
PRISMA flow diagram of the literature search, with itemized reasons for full-text exclusion.

The systematic literature search across PubMed, CINAHL, and PsycINFO databases, combined with supplementary hand-searching, yielded a total of 827 citations. After removal of duplicates, 424 unique records remained for title and abstract screening. Following review of titles and abstracts against the inclusion and exclusion criteria, 57 records were retained for full-text assessment. Then, 46 articles were excluded at the full-text stage for the following pre-specified reasons, corresponding to the exclusion criteria defined in Section 2.2: exclusive focus on healthcare providers or professional caregivers rather than family members (*n* = 10); participants who were institutional rather than family caregivers (*n* = 9); interventions examined without assessment of resilience or related outcomes (*n* = 22); and review articles, opinion pieces, or other non-empirical publications (*n* = 5). Ultimately, 11 studies met the inclusion criteria and were included in the integrative review. The PRISMA flow diagram depicting the study selection process, including the specific reasons for exclusion at the full-text stage, is presented in Figure 1.

### 3.2. Study Characteristics

The 11 included studies were published between 2017 and 2024, reflecting recent and contemporary evidence regarding caregiver resilience and post-traumatic growth. Studies were conducted across diverse geographical contexts, including Asia (4 studies conducted in China, Iran, and Nigeria), Europe (4 studies conducted in Norway, Spain, and Sweden), North America (1 study in Canada), Africa (1 study in South Africa), and international context (1 study with an international scope), providing international perspectives on the caregiving experience. Total sample size across all 11 studies was 1361 participants, with individual study samples ranging from 7 to 365 participants. The demographic characteristics of participants varied across studies; however, the majority of caregivers were female (approximately 65–70% across studies), with a mean age ranging from 40 to 65 years. Caregiver relationships to care recipients included spouses (approximately 30–35%), adult children (approximately 25–30%), siblings (approximately 15–20%), and parents (approximately 10–15%).

Regarding study design, the included studies employed diverse methodologies reflective of an integrative review approach. Five studies employed quantitative designs, including cross-sectional surveys (4 studies: Akinwale et al. [27], Mihan et al. [10], Mirhosseini et al. [24], Wu et al. [25]), and randomized controlled trials (1 study: Stjernswärd & Hansson [26]). Six studies employed qualitative approaches, including thematic analysis (2 studies: Estradé et al. [32], Villena Jimena et al. [30]), hermeneutic secondary analysis (1 study: Kalhovde & Kitzmüller [6]), in-depth interviews (2 studies: Maina et al. [28], Peng et al. [29]), and interpretative phenomenological analysis (1 study: Selotole et al. [31]). No studies (0%) employed a mixed methods design. Table 1 summarizes the study characteristics, participant demographics, designs, primary findings, and the method used to assess resilience, coping, or post-traumatic growth in each study; the corresponding MMAT quality appraisal score for each study is reported separately in Table 2 to avoid duplicating this information across tables.

### 3.3. Sources of Caregiver Stress and Burden, and Appraisal Responses

Consistent with the review’s first research question, several included studies characterized the primary sources of stress and burden experienced by family caregivers, as well as the ways in which caregivers appraised and responded to these stressors. Quantitative evidence indicated that caregiver burden in this population is substantial and comparable in magnitude between SMI and SUD caregiving contexts; Mihan et al. [10] reported that burden in caregivers of individuals with substance use disorders was statistically as high as burden in caregivers of individuals with severe mental disorders, with most caregivers across both groups reporting moderate to severe burden. Qualitative accounts converged on a small number of recurring stressors. Kalhovde and Kitzmüller [6] caregivers’ emotional trajectories as resembling an unpredictable rollercoaster, in which fear of self-harm or violence directed at or by the care recipient constituted a primary and recurring source of acute distress. Similarly, Villena Jimena et al. [30] identified an experience they termed ‘emotional storming,’ encompassing the disruption of caregivers’ own life projects and pronounced shifts in family roles and responsibilities. Maina et al. [28] documented a related but distinct appraisal, describing caregivers of individuals with substance use disorders as living in a state of perpetual crisis accompanied by anticipatory grief, which the authors termed the ‘social death’ of the relative. Selotole et al. [31] likewise characterized caregiving for a relative with substance-induced psychosis as a fundamentally destabilising responsibility.

In terms of appraisal and response, caregivers across the included studies did not appear to experience these stressors passively. Rather, they actively appraised the caregiving situation and mobilized a range of coping responses, including problem-focused strategies such as seeking information and formal support [28], emotion-focused strategies such as acceptance and religious coping [31], and meaning-focused strategies involving reframing of the caregiving role as an expression of family commitment [29]. This pattern of active appraisal and response, occurring alongside substantial and sustained burden, provides the empirical foundation for the resilience and post-traumatic growth processes addressed under the review’s second research question, discussed in the sections that follow.

### 3.4. Quality Assessment

Study quality was assessed using the MMAT version 2018. Across the 11 included studies, 1 study (9.1%) was rated as high quality (meeting all five MMAT criteria), 7 studies (63.6%) were rated as moderate quality (meeting four criteria), and 3 studies (27.3%) were rated as low quality (meeting three or fewer criteria). The high-quality study was Stjernswärd & Hansson (2017) [26], which employed a randomized controlled trial design with transparent reporting of methodological decisions. The moderate-quality studies (7 studies; 63.6%) generally demonstrated appropriate methodology with minor limitations in areas such as detailed reporting of data analysis procedures or consideration of alternative explanations for findings. The low-quality studies (3 studies; 27.3%) had more substantive methodological limitations including small sample sizes without power analysis justification [6,28,31] or limited depth of methodological description. Detailed quality assessment scores for each study are presented in Table 2. The thematic distribution of findings across the four primary domains and all 11 included studies is presented in Table 3.

### 3.5. Individual-Level Protective Factors

Addressing the review’s second research question concerning the individual, familial, social, and contextual factors that facilitate resilience and post-traumatic growth, several individual-level factors appeared to play an important role in supporting resilience and post-traumatic growth among family caregivers of people with mental illness and substance use disorders. Two studies examined emotional intelligence as a protective factor. Akinwale et al. [27] identified emotional intelligence as a significant predictor of caregiver psychological well-being, accounting for 26.2% of variance in well-being scores, with emotional intelligence enabling caregivers to manage stress effectively, recognize and regulate emotions, and communicate more adaptively with care recipients and healthcare providers [24,27]. Optimism and meaning making emerged as additional protective factors, with studies reporting that these capacities facilitated positive adaptation and post-traumatic growth [24,29,32]. Caregivers through the reviewed studies reported that their caregiving role stimulated reflection on life priorities, enhanced appreciation for relationships, and spiritual or personal transformation.

Enhanced coping capacity emerged across multiple studies (*n* = 5) as an outcome of resilience development [25,28,29,30,32]. Caregivers employed diverse coping strategies, including problem-focused coping (seeking information, accessing resources), emotion-focused coping (mindfulness, acceptance), and meaning-focused coping (finding purpose in caregiving role). However, significant variation in coping effectiveness was noted across cultural contexts. Three studies documented that the relevance and utility of specific coping strategies are culturally rooted [6,10,29], suggesting that protective factors and adaptive responses are shaped by cultural values and social contexts. Studies with low and moderate quality ratings (*n* = 10) generally lacked detailed analysis of the mechanisms underlying individual protective factors, limiting understanding of how these factors develop or can be promoted through intervention.

### 3.6. Family and Social Resources

Family dynamics and social support structures emerged as powerful protective factors shaping resilience paths in caregivers. Two studies [29,32] identified family solidarity and interconnectedness as critical motivators sustaining caregiving across decades. Particularly in contexts where familism is culturally valued, family commitment and mutual support buffered against caregiver burden and facilitated psychological adaptation. Peng et al. [29] documented this pattern in their study of 20 long-term caregivers (20+ years of caregiving) in rural China. One qualitative study, Kalhovde & Kitzmüller [6], provided an interesting contrast, finding that siblings often exhibited greater resilience than parents caring for individuals with serious mental illness, suggesting that the ability to share burdens and navigate challenges mutually enhanced coping capacity and created opportunities for personal growth.

Peer support and professional support mechanisms benefited caregivers across multiple studies. Three studies [28,30,32] addressed that participation in support groups and peer-led interventions enabled caregivers to reduce isolation, normalize their experiences, and access practical strategies from others with similar experiences. Two studies [28,30] found that caregiver validation and affirmation of coping strategies by healthcare providers enhanced psychological well-being and supported long-term resilience. However, four studies identified substantial barriers to social support access [6,10,28,31]. Socioeconomic disparities, geographical isolation, and systemic stigma constrained support mobilization in some populations. Cultural variations in help-seeking and disclosure were reported across five studies, with some cultural contexts emphasizing family-centered support over professional intervention [6,10,29,31,32].

### 3.7. Evidence-Based Interventions Enhancing Resilience

In relation to the review’s third research question concerning evidence-based interventions and their mechanisms of effect, mindfulness-based interventions demonstrated significant connection with the enhancement of caregivers’ resilience. One randomized controlled trial [26] of a web-based mindfulness program for 151 families found improvements in mindfulness, self-compassion, and perceived stress. Participants reported that mindfulness practice enabled them to break automatic stress-response patterns, adjust emotions, and regain a sense of personal identity distinct from the caregiver role. However, the authors noted that access to digital interventions was a potential barrier in some populations, particularly in low-income or geographically isolated contexts.

Cognitive reappraisal techniques and coping-based approaches demonstrated effectiveness in enhancing caregiver resilience. One cross-sectional study using structural equation modeling found that 55.56% of the variance in post-traumatic growth was explained by positive coping and resilience [25]. This finding highlights the potential for structured psychological interventions to facilitate not only burden reduction but also positive psychological transformation in caregivers. Additionally, two qualitative studies [28,30] indicated that caregivers actively employed self-care strategies and sought support, demonstrating resourcefulness and engagement in managing the caregiving experience.

### 3.8. Structural and Systemic Factors

Structural and systemic factors strongly influenced caregiver resilience paths. Two studies emphasized the relationship between healthcare system capacity and caregiver burden [10,24]. Mihan et al. [10] documented critical caregiver burden in contexts with limited access to coordinated mental health services, suggesting that system-level support structures are essential for mitigating caregiver strain. However, significant disparities existed across geographical contexts, with caregivers in resource-limited settings reporting significant barriers to care coordination and professional support.

Systemic stigma surrounding serious mental illness and substance use disorders created additional obstacles to help-seeking and social support mobilization. Four studies [6,28,31,32] found that caregivers frequently internalized societal stigma, experienced social withdrawal, and hidden the care recipient’s condition from social networks, limiting their access to informal support. However, one qualitative study [31] indicated that religious faith and community bonds served as alternative support mechanisms in contexts where professional help-seeking was constrained by stigma.

Despite these challenges, studies highlighted positive outcomes when community resources were accessible. Two studies [29,32] noted that caregivers with strong community connections reported enhanced adaptation over the caregiving path. Moreover, multiple studies [28,29,30] emphasized the importance of systemic recognition of caregiver contributions and the establishment of support services for sustaining long-term caregiving resilience.

### 3.9. Synthesis Summary

Across the 11 included studies, resilience and post-traumatic growth in family caregivers emerged as multi-dimensional constructs shaped by dynamic interactions among individual characteristics, family and social resources, intervention mechanisms, and structural contexts. Individual protective factors such as emotional intelligence (26.2% variance explained), optimism, and meaning making were consistently associated with enhanced well-being across studies [24,27,29,32]. However, these factors appeared to depend on access to supportive relationships, culturally compatible interventions, and systemic resources. High and moderate-quality studies (8 studies; 72.7% of the review) provided stronger evidence for specific protective factors and intervention mechanisms [10,24,25,26,27,29,30,32], while low-quality studies (3 studies; 27.3%) contributed important qualitative insights regarding lived experiences but lacked detailed methodological rigor [6,28,31].

Notably, significant heterogeneity was observed across cultural contexts, with protective factors and intervention effectiveness varying based on cultural values, healthcare system, and social attitudes toward mental illness and caregiving. The quality of the included studies also has implications for how confidently these findings can be interpreted. Using the MMAT, one study (9.1%) was rated high quality, seven (63.6%) moderate quality, and three (27.3%) low quality. The three low-quality studies all used qualitative designs with small samples and limited methodological detail, which means their findings, while rich in lived experience, should be treated with some caution. Nevertheless, these studies offered perspectives on caregiver experiences that quantitative studies cannot capture, particularly around emotional burden and cultural coping practices. The moderate-quality studies generally had rigorous methodology but fell short in areas such as accounting for confounding factors or providing sufficient detail about their analysis procedures. This is a common limitation in cross-sectional survey designs, which made up most quantitative studies in this review. Importantly, the findings from moderate and high-quality studies were broadly consistent with those from lower-quality studies, which gives some confidence that the overall picture presented in this review is reasonably reliable. However, the review includes only one randomized controlled trial, which is the strongest design for testing intervention effectiveness. This means that while mindfulness and cognitive reappraisal approaches show promise, the evidence base for recommending them in practice remains limited [35]. Readers and clinicians should therefore treat the intervention findings as early-stage evidence that points toward promising directions, rather than firm conclusions about what works. This variation underscores the importance of culturally adapted approaches to supporting caregivers, as no single intervention model can address the diversity of contexts in which caregiving takes place.

## 4. Discussion

### 4.1. Summary of Findings

This integrative review synthesized 11 studies (*n* = 1361 participants) examining resilience and post-traumatic growth in family caregivers of individuals with serious mental illness and substance use disorders. Across the included studies, caregiver resilience emerged as a multi-dimensional phenomenon shaped by individual protective factors, family and social resources, the availability of evidence-based interventions, and broader structural conditions. The identification of positive psychological transformation alongside burden aligns with emerging literature challenging traditional deficit-focused conceptualizations of caregiving. Notably, resilience manifestations vary significantly across cultural contexts, reflecting the embedded nature of adaptation and well-being [36].

However, it should be acknowledged that the evidence base gathered here is not evenly distributed across the two caregiving populations named in the review’s title. The majority of included studies concerned caregivers of relatives with serious mental illness, most commonly psychosis, schizophrenia, and bipolar disorder [6,26,27,29,30,32] while evidence specific to caregivers of individuals with substance use disorders was comparatively light, represented primarily by Maina et al. [28] and, in a directly comparative capacity, by Mihan et al. [10]. Two further studies occupied a conceptual middle ground, examining caregivers of individuals with substance-induced psychiatric conditions such as cannabis-induced psychosis and substance-induced psychosis disorder [27,31], which share features of both caregiving contexts without being reducible to either. Where direct comparison was possible, Mihan et al. [10] found that caregiver burden in substance use disorder contexts was statistically comparable to that observed in severe mental disorders, suggesting that the burden does not differ greatly between the two populations. However, the qualitative evidence from Maina et al. [28] pointed to caregiving experiences with a distinct phenomenological character in the SUD context, including a sense of continuous crisis and anticipatory grief that the authors termed the “social death” of the relative, alongside a heavier reliance on structured mutual-aid frameworks rather than the family-solidarity- and faith-based coping strategies that featured more in the SMI-focused studies [13,29,31]. Regarding the limited number of SUD-specific studies, this review cannot confirm whether protective factors such as emotional intelligence, optimism, and family connectedness operate similarly across both caregiving populations, or whether SUD caregiving instead requires different resilience-supporting resources, particularly structured peer support and relapse-specific coping strategies. The imbalance in the evidence base is treated as a substantive limitation of the current synthesis and is returned to in Section 4.8 and Section 4.9.

### 4.2. Methodological Quality and Its Implications

The methodological quality of studies in this review has direct implications for how confidently we can draw conclusions and make recommendations. While the overall quality was acceptable, with most studies rated moderate to high quality, several important points are worth noting. First, the dominance of qualitative designs (six out of eleven studies) reflects the relatively early stage of research in this specific area. Qualitative approaches are well-suited to exploring how caregivers experience resilience and post-traumatic growth [25], and they provided rich, contextually grounded insights in this review. However, they cannot tell us how common these experiences are, how strong the associations between protective factors and resilience are, or whether specific interventions work. This limits how far the findings can be generalized to broader caregiver populations. Furthermore, although mixed-methods designs were explicitly eligible under the inclusion criteria, none of the 11 studies identified through the search employed such an approach. This absence is unlikely to indicate a genuine methodological gap in caregiver resilience research: studies to date have tended to adopt either a qualitative, experience-focused lens or a quantitative, variable-focused lens, with little integration of the two. This is a meaningful limitation for the evidence base as a whole, since mixed-methods designs are particularly well suited to capturing the subjective, meaning-making processes underlying resilience and post-traumatic growth alongside their measurable associations and prevalence, and their absence here constrains the depth of implication that can be drawn from any single included study.

Second, the four cross-sectional quantitative studies, while providing useful data on associations between resilience and factors such as emotional intelligence and optimism, cannot establish whether these factors cause better outcomes or simply co-occur with them. This is an important distinction for practice, because recommending an intervention based on a cross-sectional association carries the risk of targeting something that may not actually drive change. Third, and perhaps most importantly, only one randomized controlled trial was included in this review [26]. While this study was rated high quality and provided encouraging findings on mindfulness-based intervention, a single trial is not sufficient to form the basis of strong clinical recommendations [37]. This gap in the evidence base is one of the most pressing priorities for future research in this field. Finally, three studies were rated low quality, primarily due to small sample sizes and limited methodological transparency. While their findings aligned broadly with those from higher-quality studies, which adds some confidence to the overall conclusions, they should not be relied upon independently to inform practice or policy decisions. Overall, given the limitations in study quality, the findings of this review are better seen as a starting point for further research than as a firm guide for clinical practice. Mental health nurses and other clinicians can draw on these findings to inform their thinking and clinical judgement but should remain aware that the evidence base is still developing.

### 4.3. Individual-Level Protective Factors

The included studies identified emotional intelligence, optimism, and meaning-making as critical individual-level protective factors. One included study found that emotional intelligence accounted for 26.2% of variance in caregiver well-being, a substantial effect size consistent with broader literature demonstrating that emotion regulation is fundamental to mental health and resilience across diverse populations [38]. Neuroscientific research has reported that emotion regulation capacities are associated with adaptive neural development and resilience paths [39]. The reviewed studies documented that caregivers employ diverse coping strategies spanning problem-focused, emotion-focused, and meaning-focused approaches. Existing stress and coping theory emphasize that effective coping is flexible and context-responsive, varying based on situational demands and cultural contexts [40]. This finding highlights the importance of culturally responsive intervention approaches rather than standardized coping strategies.

### 4.4. Social and Familial Resources

The reviewed studies emphasize that family connectedness and social support structures serve as powerful protective factors shaping caregiver resilience. Socio-ecological and family systems theories suggest that individual well-being is embedded within relational and family contexts [41]. The social determinants of health framework similarly highlight how family and community structures are fundamental determinants shaping individual health outcomes [42]. The included studies addressed that peer support and professional validation significantly benefit caregivers, findings consistent with literature demonstrating the effectiveness of peer support interventions in reducing isolation and facilitating normalization of experiences [43]. However, substantial barriers to social support access including socioeconomic disparities, geographical isolation, and systemic stigma were identified. Research on health inequities documents how structural barriers constrain support access for marginalized populations despite theoretical availability of resources [44].

### 4.5. Evidence-Based Interventions

This review identified mindfulness-based interventions and cognitive reappraisal as effective approaches for enhancing caregiver resilience. Regarding mindfulness interventions, neuroscientific research demonstrates that mindfulness practices alter neural patterns associated with emotional reactivity and self-referential processing [45]. Consistent with the findings presented in this review, randomized controlled trials of mindfulness-based stress reduction across diverse populations have documented improvements in emotional regulation and quality of life [46]. The finding that cognitive reappraisal explains substantial variance in post-traumatic growth aligns with stress-related growth theory, proposing that meaning-making and reframing of challenges facilitate positive psychological transformation [47].

### 4.6. Structural and Systemic Factors

The reviewed studies underscore that structural and systemic context’s structure caregiver resilience in ways that individual-level interventions alone cannot address. The capacity of the healthcare system, care coordination, and service accessibility all directly influence caregiver burden and outcomes. The Stress Process Model and ecological systems perspectives emphasize that individual adaptation occurs within nested institutional and social contexts [48]. Systemic stigma surrounding mental illness and substance use disorders creates critical barriers to help-seeking and social support mobilization. Research on stigma and mental health demonstrates that internalized stigma constrains help-seeking behaviors and limits access to professional support and informal social networks [49]. Anti-stigma initiatives and public education campaigns have documented effectiveness in reducing stigma and improving help-seeking [50]. Policy-level support, financial assistance, and caregiver-centered healthcare policies are associated with improved caregiver outcomes [51].

### 4.7. Cultural Heterogeneity and Adaptation

Resilience manifestations and protective factors varied significantly across cultural contexts. Studies from collectivist contexts emphasize family values and interconnectedness, while studies from individualist contexts emphasize personal agency and identity maintenance. Recent research demonstrates that constructs such as resilience, well-being, and coping are shaped and demonstrated differently across cultural contexts [52]. The study addressed that cultural differences influence whether people turn to professionals or informal networks for help, as well as how willing they are to share personal information. This finding is consistent with existing research demonstrating that culture plays a fundamental role in shaping these choices [53]. Effective caregiver support requires cultural humility, recognition of the limitations of one’s own cultural perspective and openness to learning from caregivers regarding their values and cultural strengths [54]. Research on cultural adaptation demonstrates that flexibly adapting evidence-based interventions to align with community values enhances effectiveness and engagement [55].

### 4.8. Strengths and Limitations

Strengths include the following: (1) diverse methodologies providing multiple perspectives; (2) rigorous systematic methodology with quality assessment; (3) international scope providing global perspective; (4) strengths-based focus on resilience and growth. Limitations include the following: (1) modest sample size (*n* = 11) limiting generalizability; (2) three low-quality studies, though sensitivity analyses showed consistent findings; (3) publication bias from English-language studies only; (4) heterogeneity in designs limiting quantitative synthesis; (5) limited comparative data across cultural contexts; and (6) restriction of the electronic search to three databases, namely PubMed, CINAHL, and PsycINFO. These databases were selected for their strong coverage of the nursing, health sciences, and psychology studies from which most caregiver resilience research originates; however, this choice excluded Embase, the Cochrane Library, and Web of Science, and may consequently have introduced an element of retrieval bias, particularly given that caregiver resilience scholarship also extends into social work and family medicine studies that are less comprehensively indexed within the databases searched here. Future reviews on this topic would benefit from a broader, multidisciplinary search strategy to capture this literature more comprehensively. Formal certainty of evidence assessment using frameworks such as GRADE was not conducted, as this review employed narrative thematic synthesis across heterogeneous study designs rather than meta-analysis; the confidence with which conclusions can be drawn is instead addressed through the MMAT quality assessment scores reported for each included study.

### 4.9. Future Research Directions

This review has highlighted several important gaps that future research should address. Foremost among these is the need for more rigorously designed randomized controlled trials testing interventions aimed at building resilience and supporting post-traumatic growth in family caregivers of people with serious mental illness and substance use disorders. Only one RCT was included in this review, and while its findings were encouraging, a single trial is simply not enough to draw firm conclusions about what works and for whom [37]. Future trials should include larger samples, active control conditions, longer follow-up periods, and clear reporting of how the intervention was delivered and by whom. Alongside this, there is a need for more longitudinal research that tracks how caregiver resilience develops and changes over time. All the quantitative studies in this review used cross-sectional designs, which means we can say that certain factors are associated with resilience, but we cannot say whether they lead to better outcomes over time [25]. Longitudinal studies would help us understand the caregiving journey more fully, including how resilience fluctuates during periods of crisis, remission, and relapse in the care recipient.

The mechanisms through which resilience develops also deserve closer attention. Several studies in this review identified factors associated with resilience, such as emotional intelligence, optimism, and family solidarity, but few explored how or why these factors make a difference. Understanding the underlying mechanisms would support more targeted interventions rather than simply adding components to an already complex support package [56]. Cultural context is another area that warrants dedicated research attention. This review included studies from diverse geographical settings, and the findings consistently showed that resilience looks different across cultures and that interventions need to be adapted accordingly [6,29]. However, there is currently very little research on how to adapt evidence-based caregiver interventions for specific cultural contexts in a systematic and rigorous way [57]. Future research should engage communities directly in the co-design of culturally appropriate support programs.

There is also a need for research that specifically examines the role of mental health nurses in supporting caregiver resilience. While this review points to several ways nurses could contribute, from resilience assessment to facilitating peer support and delivering psychoeducation, there is little empirical evidence directly testing nurse-led approaches in this population [58]. Studies examining the effectiveness, feasibility, and acceptability of nurse-led caregiver resilience interventions would be a valuable contribution to the field. Finally, future research should pay closer attention to caregivers who are currently underrepresented in the literature, including male caregivers, older caregivers, caregivers from lower-income settings, and those caring for individuals with co-occurring mental illness and substance use disorders. A more inclusive research agenda would ensure that support strategies are relevant and accessible to the full diversity of people taking on this important and often invisible role.

### 4.10. Implications for Practice and Policy

#### 4.10.1. Implications for Mental Health Nursing Practice

The findings of this review carry several practical messages for mental health nurses who work with family caregivers of individuals with serious mental illness and substance use disorders. The most important shift is to move away from viewing caregivers primarily as individuals who are struggling, and instead to recognize the strengths and resilience they already bring to their role. Mental health nurses are often the first point of contact for families navigating these challenges, and this puts them in a good position to assess not just burden but also coping capacity, meaning-making, and social support resources [59]. In routine practice, mental health nurses could use brief validated tools to assess caregiver resilience alongside burden, helping to build a more complete picture of how a caregiver is managing [60]. Where resilience resources appear limited, nurses can play an active role in strengthening them, for example, by facilitating access to peer support groups, encouraging mindfulness-based self-care, or providing psychoeducation that helps caregivers understand and reframe their experiences [61]. The finding that emotional intelligence accounted for 26.2% of variance in caregiver well-being [27] suggests that interventions targeting emotion regulation skills may be particularly worthwhile, and these can be delivered by nurses within routine family meetings or care planning sessions. Group-based formats deserve consideration, as several studies in this review suggested that sharing experiences with others in similar situations helped caregivers feel less alone and more confident in their coping [28]. Mental health nurses working in community or inpatient settings could advocate for and facilitate caregiver support groups as a standard part of family-centered care, rather than treating them as an optional extra [62]. Cultural sensitivity is also essential. The review found that what helps caregivers cope varies considerably across cultural contexts, with some placing greater value on family solidarity and religious faith while others draw more on personal activity and professional support [29,31]. Mental health nurses should explore what resilience means to each individual caregiver and design their support accordingly, rather than applying a one-size-fits-all approach [63].

#### 4.10.2. Implications for Health Policy

At a policy level, there is a clear need for mental health systems to formally recognize family caregivers as key partners in care, not just informal helpers who operate in the background [64]. This means building caregiver assessment and support into standard mental health service pathways, providing adequate funding for caregiver-focused interventions such as respite care, psychoeducation programs, and peer support networks, and ensuring that mental health nurses have the time and training to deliver family-centered care effectively [62]. Stigma around mental illness and substance use disorders remains a significant barrier, with several studies in this review showing that caregivers often withdraw from social support because they fear judgment from others [28,32]. Anti-stigma campaigns and public education efforts that specifically address the experiences of family caregivers could help reduce this isolation and make it easier for caregivers to seek help [65]. Healthcare system reforms that prioritize care coordination and clear referral pathways for caregiver support are also needed, particularly in under-resourced settings where caregivers currently have little access to professional help [10]. Finally, digital interventions such as web-based mindfulness programs show potential support for reaching caregivers who cannot easily access face-to-face support, though the evidence base remains limited and the quality of digital content matters as much as accessibility [66]. Policy decisions about funding digital caregiver support should be guided by outcome evidence rather than assumptions about reach and scalability alone.

## 5. Conclusions

This integrative review set out to answer three research questions concerning family caregivers of individuals with serious mental illness and substance use disorders: the primary sources of caregiver stress and burden and how caregivers appraise and respond to them; the individual, familial, social, and contextual factors that facilitate resilience and post-traumatic growth; and the evidence-based interventions that enhance resilience and psychological well-being, together with their mechanisms of effect. Synthesizing 11 studies, this review found that caregivers experience substantial and sustained burden arising from unpredictable crises, role disruption, and anticipatory grief yet actively appraise and respond to these stressors through problem-, emotion-, and meaning-focused coping (Research Question 1). Resilience and post-traumatic growth were found to be multi-dimensional outcomes shaped by interacting individual protective factors (emotional intelligence, optimism, and meaning-making), family and social resources (family solidarity, and peer and professional support), and structural conditions such as healthcare system capacity and stigma (Research Question 2). Evidence-based interventions, particularly mindfulness-based approaches and cognitive reappraisal techniques, showed encouraging but currently limited evidence of effectiveness, resting on a single randomized controlled trial and cross-sectional associations that cannot establish causal mechanisms (Research Question 3). While caregivers experience significant burden, many develop remarkable resilience and positive psychological transformation, although resilience displays vary across cultural contexts, emphasizing that adapted and context-responsive approaches are essential. Clinical practice, policy, and research should shift from deficit-focused to strengths-based approaches while addressing structural barriers, including stigma, healthcare inequities, and resource limitations, and while building a stronger evidence base, particularly through additional randomized controlled trials, before firm recommendations regarding specific interventions can be made. This integrative review contributes to a better understanding of caregiver resilience and offers evidence-based directions for advancing caregiver-focused mental health services.

## Figures and Tables

**Table 1:** Summary of the Included Studies (*n* = 11).

Author and Year	Location	Objective	Participants	Research Design	Main Findings	Resilience Points	Resilience/Coping/PTG Assessment Method
Akinwale et al. (2024) [27]	Lagos, Nigeria	To explore how emotional intelligence (EI) influences the psychological well-being of caregivers of individuals with cannabis-induced psychosis.	100 family and non-family caregivers.	Descriptive cross-sectional survey.	EI significantly impacts well-being (26.2% variance), and collectively with distress and burden, explains 52.6% of well-being scores.	High emotional intelligence acts as a protective factor, allowing caregivers to manage stress effectively and communicate to prevent burnout.	GHQ-12, ZBI, TEIQue-SF, PWB Scale (no dedicated resilience/coping/PTG instrument)
Estradé et al. (2023) [32]	International Review	To identify key experiential themes from first-person accounts of psychosis caregiving.	192 participants (including 159 caregivers).	Bottom-up thematic review of 48 first-person account sources.	Caregivers navigate a journey from initial shock to building resilience and hope despite facing stigma and loss.	Resilience is built by adjusting expectations, finding purpose or meaning in the experience	No standardized instrument; thematic analysis of 48 published first-person accounts
Kalhovde & Kitzmüller (2023) [6]	Norway	To understand the lived experiences of emotional trajectories of distress in caregivers over time.	7 family caregivers (mothers, father, spouse, siblings).	Secondary analysis using a hermeneutic approach.	Trajectories resemble an unpredictable rollercoaster; fear of self-harm and violence are primary stressors.	Siblings often exhibited greater resilience than parents due to an ability to share challenges and find opportunities for personal growth.	No standardized instrument; hermeneutic secondary analysis of interview transcripts (*n* = 7)
Mihan et al. (2023) [10]	Tehran, Iran	To investigate caregiver burden in severe mental disorders versus substance use disorder.	260 patients and their primary caregivers.	Cross-sectional study.	Burden in substance use is statistically as high as in severe mental disorders; most caregivers report moderate to severe burden.	Identifying specific burden-related factors (like patient comorbidity) is the first step for planning targeted support strategies.	Zarit Burden Interview (ZBI) only; no resilience/coping/PTG instrument administered
Maina et al. (2021) [28]	Saskatchewan, Canada	To explore experiences of caring for a relative with SUD and self-care strategies employed.	21 participants (mostly women).	Exploratory qualitative design.	Caregivers experience perpetual crisis and “social death” of the relative, but actively seek ways to mitigate impact.	Caregivers exhibit resourcefulness by attending self-help groups (Al-Anon), counseling, and seeking online support to manage fatigue.	No standardized instrument; semi-structured interviews, exploratory thematic analysis
Mirhosseini et al. (2024) [24]	Mashhad, Iran	To examine the relationship between burden, resilience, and optimism in family caregivers.	209 family caregivers.	Cross-sectional design.	80.9% reported low resilience; optimism was the strongest predictor of lower burden.	Optimism and resilience are internal resources that improve life quality and act as buffers against high caregiving stress.	ZBI; Connor-Davidson Resilience Scale (CD-RISC); Revised Life Orientation Test (LOT-R)
Peng et al. (2022) [29]	Rural China	To explore long-term caregiving (20+ years) in terms of both positive and negative aspects.	20 family caregivers.	Qualitative study using in-depth interviews.	Burden is heavy, but cultural values like familism provide a motivation to maintain care over decades.	Family solidarity acts as a motivator; recognizing the value of affection and compassion fosters growth and resilience.	No standardized instrument; semi-structured interviews, four-phase thematic analysis
Selotole et al. (2022) [31]	Giyani, South Africa	To describe experiences of caring for a relative with substance-induced psychosis disorder (SIPD).	8 family members.	Qualitative research using interpretative phenomenological analysis (IPA).	Caregiving is a destabilising responsibility; support is found through community bonds and religious practice.	Acceptance and religious coping (prayer) are the most powerful strategies used to absorb pressure and find internal solace.	No standardized instrument; in-depth telephonic interviews, interpretative phenomenological analysis
Stjernswärd & Hansson (2017) [26]	Sweden	To explore the effectiveness of a web-based mindfulness program for families.	151 families.	Randomized controlled trial (RCT).	Significant improvements in mindfulness and self-compassion; decreased levels of perceived stress.	Mindfulness training helps caregivers break automatic reactions and regain a personal identity separate from the role of “caregiver”.	Five Facet Mindfulness Questionnaire (FFMQ); Self-Compassion Scale-Short Form (SCS-SF); Perceived Stress Scale (PSS); CarerQoL7-D
Villena Jimena et al. (2024) [30]	Malaga, Spain	To explore perceptions regarding emotional impact and social functioning repercussions.	28 participants in four focus groups.	Descriptive qualitative study using thematic analysis.	Themes identified include emotional storming, disruption of life projects, and changes in family roles.	Validation of the caregiver’s experience and reaffirming personal strategies are essential for sustaining the long-term burden.	No standardized instrument; focus group interviews, thematic analysis
Wu et al. (2021) [25]	Jinan, China	To test the mediating roles of coping and resilience between social support and posttraumatic growth (PTG).	365 primary caregivers.	Cross-sectional study using SEM	Positive coping and resilience explained 55.56% of the variance in PTG; social support is a direct catalyst.	Resilience acts as the critical mediator that allows caregivers to “bounce back” and transition to growth.	Posttraumatic Growth Inventory (PTGI); Connor-Davidson Resilience Scale (CD-RISC); Simplified Coping Style Questionnaire (SCSQ); Perceived Social Support Scale

**Table 2:** MMAT Quality Assessment Scores for Included Studies (*n* = 11).

Author (Year)	Design Type	S1	S2	C1	C2	C3	C4	C5	Overall Score	Remarks
Qualitative studies *Criteria: 1.1 Appropriate approach, 1.2 Adequate data collection, 1.3 Findings derived from data, 1.4 Interpretation substantiated, 1.5 Coherence*										
Estradé et al. (2023) [32]	Qualitative	Y	Y	Y	Y	Y	Y	N*	4/5—Moderate *	Synthesis of pre-existing accounts from a broader project, not this review’s specific aim
Kalhovde & Kitzmüller (2023) [6]	Qualitative	Y	N*	Y	Y	Y	N*	N*	3/5—Low *	Secondary analysis of single interviews (*n* = 7) from an earlier study with a different aim
Maina et al. (2021) [28]	Qualitative	Y	Y	Y	Y	Y	N*	N*	3/5—Low *	Small sample (*n* = 21); limited male representation (*n* = 4)
Peng et al. (2022) [29]	Qualitative	Y	Y	Y	Y	Y	Y	N*	4/5—Moderate	Sample drawn from an existing database, limited to caregivers with 20+ years of experience, which may limit representativeness.
Selotole et al. (2022) [31]	Qualitative	Y	Y	Y	Y	N*	N*	Y	3/5—Low	Section 4 integrates outside literature closely with participant accounts, making it difficult to distinguish findings that derive directly from the data
Villena Jimena et al. (2024) [30]	Qualitative	Y	Y	Y	Y	Y	Y	N*	4/5—Moderate	Single-region sample; restricted to caregivers within 5 years of diagnosis
Quantitative RCT *Criteria: 2.1 Randomization performed, 2.2 Groups comparable at baseline, 2.3 Complete outcome data, 2.4 Outcome assessors blinded, 2.5 Participants adhered*										
Stjernswärd & Hansson (2017) [26]	Quantitative RCT	Y	Y	Y	Y	Y	Y	Y	5/5—High	None, met all criteria
Quantitative non-randomized *Criteria: 3.1 Participants representative, 3.2 Measurements appropriate, 3.3 Complete outcome data, 3.4 Confounders accounted for, 3.5 Intervention consistent*										
Mihan et al. (2023) [10]	Quantitative non-randomized	Y	Y	Y	Y	Y	Y	N*	4/5—Moderate	Key confounder (family income) not included in the assessment
Wu et al. (2021) [25]	Quantitative non-randomized	Y	Y	Y	Y	Y	Y	N*	4/5—Moderate	Cross-sectional design limits causal/directional inference
Quantitative descriptive *Criteria: 4.1 Relevant sampling strategy, 4.2 Sample representative, 4.3 Measurements appropriate, 4.4 Low nonresponse bias, 4.5 Appropriate statistical analysis*										
Akinwale et al. (2024) [27]	Quantitative descriptive	Y	Y	Y	Y	Y	N*	Y	4/5—Moderate *	Purposive/convenience sampling; response rate not reported
Mirhosseini et al. (2024) [24]	Quantitative descriptive	Y	Y	Y	Y	Y	N*	Y	4/5—Moderate *	Self-report data; authors note possible response bias

**Note:** S1 = Screening question 1: Are there clear research questions? S2 = Screening question 2: Do the collected data allow to address the research questions? C1–C5 = Design-specific criteria (see section headers for full wording per design category). Y = Yes; N* = No/Cannot tell. Studies are grouped by design type. Numbers refer to study order in Table 1. **Quality categories:** High = all five criteria met (5/5); Moderate = four criteria met (4/5); Low = three or fewer criteria met (≤3/5). An asterisk (*) following the overall quality category indicates that the corresponding Remarks entry identifies a study-specific limitation affecting interpretation of that score.

**Table 3:** Thematic Distribution Across Included Studies (*n* = 11).

Author (Year)	Theme 1 Individual-Level Protective Factors			Theme 2 Family & Social Resources			Theme 3 Evidence-Based Interventions		Theme 4 Structural and Systemic Factors	
	Emotional Intelligence	Optimism and Meaning-Making	Coping Strategies	Family Solidarity	Peer and Professional Support	Barriers to Support	Mindfulness-Based	Cognitive Reappraisal and Self-Care	Healthcare System Capacity	Stigma and Community Bonds
Akinwale et al. (2024) [27]	✓	-	✓	-	-	-	-	-	-	✓
Estradé et al. (2023) [32]	-	✓	✓	✓	✓	✓	-	-	-	✓
Kalhovde & Kitzmüller (2023) [6]	-	-	✓	✓	-	✓	-	-	-	✓
Mihan et al. (2023) [10]	-	-	✓	-	-	✓	-	-	✓	-
Maina et al. (2021) [28]	-	-	✓	-	✓	✓	-	✓	-	✓
Mirhosseini et al. (2024) [24]	✓	✓	-	-	-	-	-	-	✓	-
Peng et al. (2022) [29]	-	✓	✓	✓	-	✓	-	-	-	-
Selotole et al. (2022) [31]	-	-	-	-	-	✓	-	-	-	✓
Stjernswärd & Hansson (2017) [26]	-	-	-	-	-	-	✓	-	-	-
Villena Jimena et al. (2024) [30]	-	-	✓	-	✓	-	-	✓	-	-
Wu et al. (2021) [25]	-	-	✓	-	-	-	-	✓	-	-

Note: ✓ = Theme addressed in study; - = Theme not addressed.

## Data Availability

The original contributions presented in the study are included in the article; further inquiries can be directed to the corresponding authors.

## Supplementary Materials

The following supporting information can be downloaded at: https://www.mdpi.com/article/10.3390/nursrep16080280/s1, PRISMA 2020 Checklist.

## Public Involvement Statement

There was no public involvement in any aspect of this research.

## Guidelines and Standards Statement

This manuscript was drafted using PRISMA (Preferred Reporting Items for Systematic Reviews and Meta-Analyses for Protocols) guidelines.

## Use of Artificial Intelligence

During the preparation of this manuscript, the authors used Grammarly (version not applicable, cloud-based; Grammarly Inc., San Francisco, CA, USA; https://www.grammarly.com; accessed 12 March 2026) for grammar and spelling checking, and NotebookLM (version not applicable, cloud-based; Google LLC, Mountain View, CA, USA; https://notebooklm.google.com; accessed 12 March 2026) for the purposes of verifying the reference list. The authors have reviewed and edited the output and take full responsibility for the content of this publication.

## Abbreviations

The following abbreviations are used in this manuscript:

MMAT	Mixed Methods Appraisal Tool
SMI	Serious mental illness
SUD	Substance use disorders
RCT	Randomized Controlled Trial
