# Supplementary material for: From ‘Emotional Storm’ to Post-Traumatic Growth Among Caregivers of Individuals with Mental Illness and Substance Use Disorders: An Integrative Review
Source: Nurs Rep. 2026 Aug 11;16(8):280. doi: 10.3390/nursrep16080280 (PMC13516421; doi:10.3390/nursrep16080280)
Supplement: Supplementary file 1 [file nursrep-16-00280-s001.zip › nursrep-4377100-supplementary.pdf]

PRISMA 2020 Checklist

Manuscript: "From 'Emotional Storm' to Post-traumatic Growth among Caregivers of Individuals with Mental Illness and Substance Use Disorders: An Integrative Review"

Section & Topic	Item #	Checklist item	Status	Location	Notes / explanation
TITLE	1	Identify the report as a systematic review.	Partially reported	Page 1	Manuscript is framed as an integrative review; PRISMA is used only to inform reporting, consistent with the Whittemore & Knafl framework stated in Section 2.
ABSTRACT	2	See the PRISMA 2020 for Abstracts checklist.	Reported	Page 1	
INTRODUCTION	3	Describe the rationale for the review in the context of existing knowledge.	Reported	Page 2–3	
	4	Provide an explicit statement of the objective(s) or question(s) the review addresses.	Reported	Page 4	Three explicit research questions stated at the end of the Problem Identification subsection.
METHODS	5	Specify the inclusion and exclusion criteria for the review and how studies were grouped for the syntheses.	Reported	Page 4–5	
	6	Specify all databases, registers, websites, organisations, reference lists and other sources searched or consulted. Specify the date each source was last searched.	Partially reported	Page 4	Databases and hand-searching are named; exact search date(s) are not stated. This is disclosed as a limitation of the search reporting.
	7	Present the full search strategies for all databases, registers and websites, including filters and limits.	Reported	Page 4	Boolean search string reported; database-specific filter settings not itemised.
	8	Specify the methods used to decide study eligibility, including number of reviewers, independence, and automation tools.	Reported	Page 5	Corrected from "Page 4-5." Two reviewers screened independently; disagreements resolved by discussion.
	9	Specify the methods used to collect data from reports (reviewers, independence, contact with investigators, automation).	Reported	Page 5–6	Corrected from "Page 4-5." Now explicitly describes template piloting on 2 studies and independent extraction by 2 reviewers with cross-checking (revised 2.3).

Section & Topic	Item #	Checklist item	Status	Location	Notes / explanation
METHODS (cont.)	10a	List and define all outcomes for which data were sought; state whether all compatible results were sought.	Reported	Page 5	Corrected from “Page 4-5.”
	10b	List and define all other variables for which data were sought; describe assumptions about missing/unclear information.	Reported	Page 5	Corrected from “Page 4-5.”
	11	Specify the methods used to assess risk of bias, including tool(s) used, number of reviewers, and independence.	Reported	Page 5-6	Corrected from “Page 4-5.” MMAT 2018; two reviewers independently, consensus by discussion.
	12	Specify the effect measure(s) used in the synthesis or presentation of results.	Not applicable	N/A	No meta-analysis conducted; thematic synthesis only (justified in 2.4).
	13a	Describe the processes used to decide which studies were eligible for each synthesis.	Reported	Page 6	Corrected from “Page 5-6.”
	13b	Describe methods to prepare data for presentation/synthesis (missing statistics, data conversions).	Reported	Page 6	Corrected from “Page 5.”
	13c	Describe methods used to tabulate or visually display results of individual studies and syntheses.	Reported	Page 6	Corrected from “Page 5-6.” Refers to Tables 1-3 and Figure 1.
	13d	Describe methods used to synthesise results and provide a rationale for the choice(s).	Reported	Page 6	Corrected from “Page 5.” Thematic synthesis (Braun & Clarke) rationale given explicitly.
	13e	Describe methods used to explore possible causes of heterogeneity among study results (e.g. subgroup analysis, meta-regression).	Partially reported	Page 6	Findings organised by region, culture, and disorder type (2.4); this is a narrative organisation, not a formal subgroup analysis or meta-regression.
	13f	Describe any sensitivity analyses conducted to assess robustness of the synthesised results.	Partially reported	Page 6 (method, implicit); Page 18 (3.9); Page 20 (4.2); Page 24 (4.8)	Corrected from “Page 15.” No formal a priori sensitivity-analysis method is named in 2.4; results narrative (3.9) and limitations (4.8) refer to consistency checks across quality strata rather than a defined sensitivity procedure.

Section & Topic	Item #	Checklist item	Status	Location	Notes / explanation
METHODS (cont.)	14	Describe methods used to assess risk of bias due to missing results in a synthesis (reporting biases).	Not reported (Gap)	N/A	Not formally assessed. This is acknowledged here as a limitation of the review; a dedicated methodological approach to reporting-bias assessment was not part of the review's scope.
	15	Describe methods used to assess certainty (confidence) in the body of evidence for an outcome.	Partially reported	Page 5-6 (MMAT method); Page 24-25 (4.8, explicit GRADE statement)	Corrected from "Page 21." GRADE not used; manuscript explicitly states MMAT scores serve this function instead (4.8).
RESULTS	16a	Describe the results of the search and selection process, ideally using a flow diagram.	Reported	Page 7-8	Corrected from "Page 5–7." PRISMA flow diagram (Figure 1).
	16b	Cite studies that might appear to meet inclusion criteria but were excluded, and explain why.	Not reported (Gap)	Page 7-8	Only aggregate exclusion counts by reason are given in Figure 1; individual excluded studies are not cited by name.
	17	Cite each included study and present its characteristics.	Reported	Page 7 (narrative, 3.2); Page 8-13 (Table 1)	Corrected from "Page 7, 9-12."
	18	Present assessments of risk of bias for each included study.	Reported	Page 14-15 (Table 2)	Corrected from "Page 12-13." Now includes the added "Remarks" column.
	19	For all outcomes, present summary statistics and/or effect estimates per study, ideally in structured tables or plots.	Reported	Page 8-13 (Table 1); Page 13-17 (narrative)	Corrected from "Page 9-12; Page 12-17." No effect estimates (not a meta-analysis); narrative and tabulated findings only.
	20a	For each synthesis, briefly summarise the characteristics and risk of bias among contributing studies.	Reported	Page 16-18 (3.5-3.9)	Corrected from "Page 14-17."
	20b	Present results of all statistical syntheses conducted (summary estimate, precision, heterogeneity).	Not applicable	N/A	No meta-analysis conducted.

	20c	Present results of all investigations of possible causes of heterogeneity among study results.	Partially reported	Page 15 (Table 3); Page 16-18 (narrative)	Corrected from “Page 14-18, 21.” Presented as thematic/cultural variation, not formal heterogeneity statistics.
RESULTS (cont.)	20d	Present results of all sensitivity analyses conducted to assess robustness of the synthesised results.	Partially reported	Page 18 (3.9); Page 20 (4.2); Page 24 (4.8)	Corrected from “Page 18, 21.” See note at item 13f, informal consistency check across quality strata, not a pre-specified sensitivity analysis.
	21	Present assessments of risk of bias due to missing results (reporting biases) for each synthesis assessed.	Not applicable	N/A	Consistent with item 14, not assessed.
	22	Present assessments of certainty (confidence) in the body of evidence for each outcome assessed.	Partially reported	Page 14-15 (Table 2); Page 17-20 (3.9, 4.2 narrative)	Corrected from “Page 12-14, 17-19.” Certainty conveyed via MMAT quality strata rather than a formal GRADE rating per outcome.
DISCUSSION	23a	Provide a general interpretation of the results in the context of other evidence.	Reported	Page 18-19 (4.1)	Corrected from “Page 18, 20.”
	23b	Discuss any limitations of the evidence included in the review.	Reported	Page 19-20 (4.2); Page 23-24 (4.8)	Corrected from “Page 18-19.”
	23c	Discuss any limitations of the review processes used.	Reported	Page 23-24 (4.8)	Corrected from “Page 21.”
	23d	Discuss implications of the results for practice, policy, and future research.	Reported	Page 25-27 (4.9-4.10)	Corrected from “Page 21-24.”
OTHER INFORMATION	24a	Provide registration information for the review, including register name and number, or state the review was not registered.	Reported	Not registered	Stated directly, no location correction needed.
	24b	Indicate where the review protocol can be accessed, or state that a protocol was not prepared.	Reported	No protocol	Stated directly.
	24c	Describe and explain any amendments to information provided at registration or in the protocol.	Not applicable	N/A	Not applicable, as the review was not registered and no protocol was prepared (see 24a, 24b).
	25	Describe sources of financial or non-financial support for the review, and the role of funders/sponsors.	Reported	Page 29	Corrected from “Page 24.” “Funding” statement: no external funding received.

	26	Declare any competing interests of review authors.	Reported	Page 29	Corrected from “Page 25.”
	27	Report which of the following are publicly available: data collection forms; extracted data; analytic data/code; other materials.	Reported	Page 29	Corrected from “Page 25.” Data Availability Statement: further inquiries directed to corresponding author.

From: Page MJ, McKenzie JE, Bossuyt PM, Boutron I, Hoffmann TC, Mulrow CD, et al. The PRISMA 2020 statement: an updated guideline for reporting systematic reviews. BMJ 2021;372:n71. doi: 10.1136/bmj.n71.
This work is licensed under CC BY 4.0.
